# An overview of mechanisms, biomarkers, and treatment strategies for acquired anti-EGFR resistance in RAS wild-type colorectal cancer

**DOI:** 10.3389/fphar.2025.1656372

**Published:** 2025-09-29

**Authors:** Chenxiang Ding, Ruoyu Yao, Yong Wang, Hongjie Liu, Chunyan Li, Hua Xie

**Affiliations:** ^1^ Clinical Medicine, Bengbu Medical University Graduate School, Bengbu, China; ^2^ Department of Oncology, Affiliated Xuancheng Hospital of Wannan Medical College, Xuancheng People’s Hospital, Xuancheng, China; ^3^ Department of Oncology, Anhui Xuancheng Central Hospital, Xuancheng, China

**Keywords:** colorectal cancer, acquired anti-EGFR resistance, molecular mechanism, predictive biomarker, coping strategy

## Abstract

Colorectal cancer (CRC) is a threat to public health, with a global incidence and mortality of over 1.9 million and 0.9 million people, respectively. Anti-epidermal growth factor receptor (EGFR)-based treatment is recommended for CRC with wild-type rat sarcoma (RAS). However, after continuous treatment with this regimen, acquired resistance occurs, which hampers the prognosis of patients. The median progression-free survival of patients with metastatic CRC receiving first-line anti-EGFR-based treatment is 10–13 months. The widely recognized mechanisms that induce acquired anti-EGFR resistance are related to the activation of the RAS/RAF/MEK/ERK and PI3K/AKT/mTOR signaling pathways. In addition, novel mechanisms that induce acquired resistance, such as microsatellite stability/mismatch repair status, noncoding RNAs, the tumor microenvironment, exosome-mediated intracellular communication, and post-transcriptional modification, are being discovered. To improve personalized medication, biomarkers with predictive value for acquired anti-EGFR resistance are recognized from both tumoral samples and liquid biopsies. On the basis of the identified mechanisms, clinicians have developed several treatment strategies to cope with acquired anti-EGFR resistance. This review provides an overview of acquired anti-EGFR resistance in RAS wild-type CRC by summarizing the common genes and proteins, potential novel mechanisms, and predictive biomarkers related to acquired anti-EGFR resistance, as well as treatment strategies to address this resistance. This review may serve as a potential reference for exploring possible treatment strategies for acquired anti-EGFR resistance.

## 1 Introduction

Colorectal cancer (CRC) is a prevalent tumor worldwide. According to statistics published in 2024, the annual incidence and mortality of CRC are over 1.9 million and 0.9 million, ranking third and second, respectively, among all sites of cancer (Bray et al., 2024). Although the etiology of CRC remains unclear, the risk factors for CRC have been recognized. The common risk factors for CRC include a familial history of Lynch syndrome and adenomatous polyposis coli, inflammatory bowel disease, diabetes mellitus, an excessive diet of red and processed meat, a low diet of fruit and vegetables, a sedentary lifestyle, cigarette and alcohol consumption, age, and dysbiosis of the gut microbiota (Sawicki et al., 2021). The identification of risk factors for CRC may help improve early screening strategies; however, owing to the insidious nature of early-stage CRC, many patients are at an advanced stage at the time of diagnosis (Gupta, 2022; Burnett-Hartman et al., 2021).

For advanced or metastatic CRC, the identification of genetic mutations, among which rat sarcoma (RAS) is critical, is highly important for guiding treatment (Lakatos et al., 2020; Morris et al., 2023). The RAS gene is a proto-oncogene that aids in cell growth, differentiation and signaling. When the RAS gene is mutated, it continues to activate intracellular signaling pathways, resulting in aberrant cell multiplication and differentiation, which promotes tumorigenesis and progression (Zhu et al., 2021). Epidermal growth factor receptor (EGFR) is a protein receptor expressed on the membrane of cells that is responsible for cancer progression. By binding with extracellular growth factors, EGFR can be activated, which further activates downstream signaling pathways and induces the proliferation and differentiation of tumor cells (Levantini et al., 2022). Anti-EGFR therapy is employed to impede the growth and dissemination of tumors through the use of targeted drugs against EGFR. These drugs bind to EGFR, thereby preventing activation of the EGFR signaling pathway (Kasi et al., 2023). For CRC patients harboring wild-type RAS, anti-EGFR therapy is an effective treatment that can significantly improve patient survival and prognosis (Watanabe et al., 2023; Fakih et al., 2023; J et al., 2007; Ciardiello et al., 2024; Strickler et al., 2023).

Currently, cetuximab and panitumumab are important anti-EGFR agents for treating RAS wild-type CRC. Both targeted therapeutic agents can act on the EGFR receptor and block its downstream signaling pathways, such as the RAS/RAF/mitogen-activated protein kinase (MAPK)/extracellular regulated protein kinase (ERK) pathways, to inhibit tumor proliferation and metastasis. According to the 2024 edition of the Chinese Society of Clinical Oncology (CSCO) Colorectal Cancer Treatment Guidelines, cetuximab + FOLFOX/FOLFIRI (oxaliplatin, fluorouracil, and calcium folinic acid/folic acid, 5-fluorouracil and irinotecan) is the preferred anti-EGFR regimen for wild-type RAS CRC (Wang et al., 2025). The 2022 edition of the National Comprehensive Cancer Network (NCCN) colorectal cancer guidelines also suggest that panitumumab + FOLFOX/FOLFIRI is a first-line option for wild-type RAS CRC patients (Benson et al., 2022; Benson et al., 2021). However, in RAS wild-type patients, alterations in colorectal cancer-related genes during anti-EGFR therapy can lead to drug resistance (Zhou et al., 2021; Ciappina et al., 2025).

Recent clinical trials have reported an objective response rate (ORR) of approximately 75%–85%, a median duration of response of approximately 12 months, and a median progression-free survival (PFS) of approximately 10–15 months for first-line anti-EGFR-based regimens in patients with metastatic CRC harboring wild-type RAS (Watanabe et al., 2023; Shiozawa et al., 2024; Wang et al., 2024; Pinto et al., 2024). Therefore, anti-EGFR therapy seems to be effective in most patients who are naïve to treatment. However, more than half of the patients experienced disease progression within 1 year after treatment initiation. For anti-EGFR-based regimens as second-line therapy or above, the ORR is approximately 23%–30%, and the median PFS is approximately less than 6 months (Yang et al., 2024; Boige et al., 2023; Hochster et al., 2024). These facts highlight the burden of acquired anti-EGFR resistance in patients with CRC harboring wild-type RAS. As a result, in-depth exploration of the molecular mechanism of acquired anti-EGFR resistance and potential strategies to cope with it is critical to improve the management of patients with CRC harboring wild-type RAS. Although previous reviews have revealed several potential mechanisms associated with acquired anti-EGFR resistance (Wang et al., 2022; Albadari et al., 2023), several novel perspectives, such as non-coding RNAs, post-transcriptional modifications, and exosome-mediated intracellular communication, have been proposed recently (Zhou et al., 2015; Mason et al., 2024; Yang et al., 2025).

This review presents an overview of acquired anti-EGFR resistance in RAS wild-type CRC by summarizing the common genes and proteins, potential novel mechanisms, and predictive biomarkers related to acquired anti-EGFR resistance, as well as treatment strategies to address this resistance.

## 2 Common genes and proteins related to acquired anti-EGFR resistance

### 2.1 Widely known genes related to acquired anti-EGFR resistance

Although anti-EGFR treatment is recommended for patients with wild-type RAS, patients could acquire RAS mutations with the continuous use of anti-EGFR antibodies. This is hypothesized to be the consequence of sustained stress conditions induced by anti-EGFR antibodies on tumor cells and the tumor microenvironment (Rios-Hoyo et al., 2024). According to previous studies, the prevalence of RAS mutations after anti-EGFR treatment ranges from 7.5% to 84% (Diaz et al., 2012; Morelli et al., 2015; Vidal et al., 2017; Toledo et al., 2017; Normanno et al., 2018; Pietrantonio et al., 2017; Strickler et al., 2018; Kim et al., 2018; Takayama et al., 2018; Vitiello et al., 2019; Yamada et al., 2020; Lim et al., 2021; Rachiglio et al., 2022).

The BRAF (B-Raf) proto-oncogene belongs to the RAF gene family and is a vital serine/threonine protein kinase that is part of the RAS/RAF/MAPK signaling pathway. BRAF-mediated signaling involves the binding of RAF to MAPK kinases (MAPKK/MEK1/2), which regulate cell proliferation. BRAF mutations are present only in RAS wild-type patients, and approximately 9% of patients have BRAF (V600E) mutations (Tol et al., 2009). The BRAF (V600E) mutation results in an abnormally active, upstream signal-independent BRAF protein, leading to sequential activation of downstream MAPK/MEK/ERK pathways, which contributes to acquired anti-EGFR resistance (Wu et al., 2020).

Human epidermal growth factor receptor 2 (HER2) belongs to the epidermal growth factor receptor family. The binding of HER2 with other members of the family results in the formation of heterodimers and enables autophosphorylation, which can further bind to various downstream signaling molecules and activate multiple signaling pathways. In addition, the heterodimeric form of HER2 can more readily return to the cell surface in response to multiple subsequent stimuli, which further contributes to the acquisition of drug resistance (Bronte et al., 2015). Approximately 3%–4% of mCRC patients have HER2 amplification, which is mostly observed in patients with wild-type RAS/BRAF tumors (Loree et al., 2018). Several studies have shown that HER2 gene amplification may be a critical contributor to acquired anti-EGFR resistance in mCRC and may also serve as a negative marker for predicting the response to anti-EGFR therapy in mCRC patients (Barry et al., 2016; Takegawa et al., 2016).

MET is the gene encoding the MET receptor tyrosine kinase, which can be serially activated by gene amplification, mutation or autocrine or paracrine stimuli from ligands. Activated MET further triggers several pathways, such as the phosphatidylinositol-3-kinase/protein kinase B/mammalian target of rapamycin (PI3K/AKT/mTOR), RAS/RAF/MAPK/ERK, and SRC/signal transducer and activator of transcription 3 (STAT3) pathways, to induce cell invasion and inhibit apoptosis (Comoglio et al., 2008). Bardelli et al. suggested that wild-type KRAS CRC cells exhibit high levels of MET gene amplification after the acquisition of anti-EGFR resistance (Bardelli et al., 2013). MET is overexpressed in a number of malignant tumors, including CRC, and is further enhanced in tumors refractory to EGFR signaling inhibition.

The insulin-like growth factor-1 receptor (IGF-1R) is a receptor tyrosine kinase on cell membranes that binds to IGF-1 and IGF-2. The IGF-1/IGF-1R signaling pathway is important for a variety of biological processes, including CRC proliferation, differentiation, and invasion, and its overexpression in cancer cells inhibits apoptosis by activating multiple intracellular signaling pathways (Wang et al., 2023). Hyperactivation of IGF-1R impedes drug-triggered apoptotic signaling by upregulating the PI3K/AKT pathway. This desensitization of CRC cells to anti-EGFR drugs leads to drug resistance (Vigneri et al., 2015). Scartozzi et al. reported that after anti-EGFR treatment of wild-type KRAS mCRC, patients with a poor prognosis had high expression of IGF-1 (Scartozzi et al., 2010).

Phosphatidylinositol-4,5-bisphosphate 3-kinase, catalytic subunit alpha (PIK3CA) is the p110-α catalytic subunit of PI3K, whose activation further initiates the PI3K/AKT/mTOR pathway to regulate various cell biological activities, such as proliferation, metabolism and apoptosis (Janku et al., 2018). Studies have shown that PIK3CA mutations are most commonly found in exons 20 and 9, but only PIK3CA exon 20 mutations are related to the outcome of drug resistance in wild-type KRAS CRC patients receiving anti-EGFR therapy (De Roock et al., 2010; Mao et al., 2012).

A recent study by Johnson RM and colleagues revealed that AT-rich interactive domain-containing protein 1 (ARID1A) gene mutation is significantly related to resistance to anti-EGFR treatment in CRC patients. The ARID1A gene is an epigenetic regulator located on chromosome 1. It works together with the multisubunit chromatin-remodeling complex SWI/SNF chromatin remodeling complex to regulate and remodel the chromatin structure. This modulation of gene expression and cellular function helps maintain cellular homeostasis and prevent excessive signaling pathway activation. A decrease in SWI/SNF activity after ARID1A mutation induces EGFR activation, leading to resistance to anti-EGFR drugs in CRC patients (Johnson et al., 2022).

### 2.2 Proteins related to acquired anti-EGFR resistance

PRSS is a serine protease secreted by tumor cells that promotes angiogenic and invasive behavior by degrading the extracellular matrix. PRSS is highly expressed in cetuximab-resistant CRC cells (Tan et al., 2020). Three distinct PRSSs were identified in this study, among which PRSS1, a pancreatic protease that is expressed mainly in the pancreas and involved in the digestive process, is significantly associated with cetuximab resistance. The possible mechanism is that PRSS1 impairs the inhibitory effect of cetuximab on the PI3K/AKT and MEK/ERK pathways; PRSS1 is also able to cleave monoclonal antibodies such as cetuximab, trastuzumab, and bevacizumab, thereby reducing their efficacy and ultimately leading to resistance. In addition, the team reported that serine peptidase inhibitor Kazal type 1 (SPINK1) inhibits the cleavage of cetuximab by PRSS1 and that SPINK1 in combination with cetuximab can resensitize cetuximab-resistant tumor cells (Tan et al., 2020). Therefore, pharmacological inhibition of PRSS1 may be an effective solution for patients with CRC who are resistant to anti-EGFR therapy and have high serum PRSS1 levels.

In a recent retrospective cohort study, phospholipase C γ 1 (PLCγ1) was shown to be highly expressed in CRC patients treated with cetuximab (Cruz-Duarte et al., 2022). Additionally, a high level of PLCγ1 contributes to cetuximab resistance in RAS wild-type colorectal cancer cells. Before resistance is acquired, tumor cells cannot maintain the downstream signaling pathway under cetuximab treatment; however, when PLCγ1 is overexpressed, the PLCγ1-SH2-containing protein tyrosine phosphatase 2 (SHP2) tandem structural domain activates ERK and AKT in parallel through the noncatalytic role of SHP2, thus ensuring continued activation of downstream signaling and leading to resistance to cetuximab. The study also revealed that, in combination with an SHP2 inhibitor, cetuximab exerts an antitumor effect on PLCγ1-dependent cetuximab-resistant cancer cells (Cruz-Duarte et al., 2022).

Luo’s research team reported that occult metastatic tissues from CRC patients presented an increase in methyltransferase-like protein 4 (METTL4), an enzyme that catalyzes the N6-methyladenosine (m6A) modification of RNA molecules. Moreover, METTL14-mediated m6A modification stabilizes pleckstrin homology-like domain family member 2 (PHLDB2) mRNA by preventing its degradation, leading to upregulation of the protein level of PHLDB2. The overexpression of PHLDB2 contributes to colorectal cancer cell resistance to cetuximab and CRC cell metastasis through two mechanisms. First, PHLDB2 stably binds to EGFR through the Arg1163 site, thereby promoting its nuclear translocation to continuously activate proliferative signaling. Second, PHLDB2 competes with ubiquitin for binding to EGFR to prevent EGFR degradation by ubiquitin (Luo et al., 2022). Researchers also reported that the replacement of the arginine at position 1,163 in the PHLDB2 sequence with an alanine (R1163A mutation) eliminated the ability of PHLDB2 to promote EGFR nuclear localization, and CRC cells harboring the R1163A mutation in PHLDB2 were no longer resistant to anti-EGFR drugs (Luo et al., 2022).

## 3 Potential novel mechanisms related to acquired anti-EGFR resistance

### 3.1 Microsatellite stability/mismatch repair function

After long-term anti-EGFR treatment, the microsatellite stability/mismatch repair function of CRC cells gradually decreases, and these cells can gradually exhibit a mismatch repair defective (dMMR) phenotype; in turn, these cells exhibit high microsatellite instability (MSI-H) and generate irreversible resistance to anti-EGFR drugs. The incidence of MSI-H CRC in patients is 12%–15% (Haraldsdottir, 2017), and MSI-H CRC is associated with shorter survival (Innocenti et al., 2019). MSI-H tumors are associated with many gene mutations, which can lead to the coding of antigens with a high degree of immunogenicity, potentially improving the immune response of tumor-infiltrating lymphocytes (Luchini et al., 2019). Therefore, immunotherapy is important for patients with MSI-H disease. Immune checkpoint inhibitors (ICIs), including the PD-1 inhibitors nivolumab and pembrolizumab and the anti-cytotoxic T lymphocyte-associated protein 4 (CTLA-4) monoclonal antibody ipilimumab, have achieved good results in MSI-H CRC patients (Petrelli et al., 2020).

### 3.2 Noncoding RNAs

Noncoding RNAs, which include mainly microRNAs (miRNAs), long noncoding RNAs (lncRNAs), and circular RNAs (circRNAs), lack the ability to translate into RNAs but exert vital regulatory effects on diverse biological processes (Sommerauer and Kutter, 2022). In recent years, studies have revealed that noncoding RNAs participate in the acquisition of anti-EGFR resistance in CRC. microRNAs can bind to the 3′ untranslated region (UTR) of mRNAs to modulate the translation of a specific protein, thus exerting their function. Chen et al. reported that after treatment with cetuximab, miRNA-216b was significantly downregulated, whereas Beclin-1 and its associated adaptive autophagy were significantly activated in CRC cells, which led to resistance to cetuximab treatment (Chen et al., 2016). Moreover, a bioinformatics study revealed that miRNA-216b targeted the 3′UTR of Beclin-1 mRNA to negatively regulate the translation of Beclin-1 protein expression, which was further confirmed by a luciferase reporter gene assay (Chen et al., 2016). Zhou reported that miRNA-133b was downregulated in CRC cells and that restoration of miRNA-133b inhibited the proliferation and invasion of CRC cells. Moreover, miRNA-133b targeted the 3′UTR of EGFR and negatively regulated EGFR expression; thus, miRNA-133b insufficiency led to anti-EGFR resistance. The authors further reported that the combination of a miRNA-133b mimic and cetuximab had a synergistic anti-CRC effect (Zhou et al., 2015).

Unlike miRNAs, lncRNAs and circRNAs serve as decoys of miRNAs to further exert their functions. Zhang et al. established cetuximab-resistant Caco-2 cells and reported that the lncRNA cetuximab resistance-associated RNA transcript 16 (CRART16) was upregulated in these cells. The lncRNA CRART16 serves as a decoy of miRNA-371a-5p to further regulate the downstream V-Erb-B2 Erythroblastic Leukemia Viral Oncogene Homolog 3. Moreover, lncRNA CRART16 overexpression was associated with the differential expression of genes enriched in the MAPK signaling pathway (Zhang et al., 2020). Another study reported that the circRNA interferon gamma receptor 2 (IFNGR2) promoted the proliferation, migration, and invasion of CRC cells both *in vitro* and *in vivo*. By sponging miRNA-30b, the circRNA IFNGR2 increases the mRNA expression of wild-type and mutant-type KRAS, which further activates the AKT signaling pathway. Moreover, the overexpression of the circRNA IFNGR2 reduces the sensitivity of CRC cells to cetuximab treatment both *in vitro* and *in vivo* (Zhang et al., 2023).

### 3.3 Tumor microenvironment

The tumor microenvironment has an important impact on the processes of tumorigenesis, development, invasion and metastasis and is also an important target for tumor therapy (Xiao and Yu, 2021). Cancer-associated fibroblasts (CAFs) and inflammatory cytokines are the two main factors that mediate cetuximab resistance in the tumor microenvironment. CAFs are the predominant stromal cell type in the tumor microenvironment, accounting for up to 50%–90% of the total cells. CAFs can promote tumor cell growth, invasion and metastasis by secreting a variety of biologically active molecules, such as growth factors, cytokines and proteases (Biffi and Tuveson, 2021). Garvey CM et al. reported that colorectal cancer patients have increased CAF abundance in the tumor microenvironment after anti-EGFR treatment. Moreover, CAFs secrete exogenous epidermal growth factor (EGF) to maintain the conduction state of the MAPK signaling pathway under anti-EGFR therapy, leading to drug resistance in CRC cells (Garvey et al., 2020).

Inflammatory cytokines in the tumor microenvironment also mediate resistance to anti-EGFR drugs. The inflammatory cytokines interleukin-1α (IL-1α), IL-1β, and IL-8 and the activated inflammatory transcription factor NF-κB are responsible for the activation of signaling pathways that overcome the drug response and lead to tumor cell resistance after anti-EGFR therapy, and these cytokines may reduce the response of other sensitive cells to cetuximab through paracrine or autocrine effects (Gelfo et al., 2016). Inhibition of IL-1 receptor signaling via the use of the recombinant decoy TRAP IL-1 decreased CRC cell proliferation and inactivated the MAPK/AKT pathway, suggesting the potential of inhibiting inflammatory cytokine signaling as a strategy for reversing anti-EGFR resistance (Gelfo et al., 2018).

### 3.4 Exosome-mediated intracellular communication

Exosomes are extracellular vesicles secreted by all cells and have diameters ranging from 40 to 100 nm; these vesicles contain DNA, RNA, proteins, and lipids as cargos. Cells can also absorb exosomes and simultaneously absorb the cargo contained in the exosomes. In recent years, exosomes have received increasing interest, and exosome-mediated intracellular communication has been shown to participate in various biological processes (Tang et al., 2025). Studies have also shown that exosome-mediated intracellular communication plays a vital role in acquired resistance to anti-EGFR treatment in CRC. A study conducted by Mason et al. revealed that under nutrient stress, cetuximab-resistant, KRAS-mutant CRC cells released amphiregulin-carrying exosomes with the monomeric G protein Rab11a. When receiving amphiregulin-carrying exosomes with the monomeric G protein Rab11a, KRAS wild-type CRC cells acquired resistance to cetuximab. However, free amphiregulin had no such effect, suggesting the role of exosome-mediated intracellular communication in acquired anti-EGFR resistance (Mason et al., 2024). Wei et al. reported that exosomes derived from multidrug-resistant CRC cells induced resistance to cetuximab in CRC cells that were previously sensitive to cetuximab through the activation of the PI3K/AKT signaling pathway. Moreover, exosomes derived from multidrug-resistant CRC cells promoted the stemness of cetuximab-sensitive CRC cells. Similar findings were also reported in xenograft models (Wei et al., 2023). A study conducted by Morimura et al. reported that exosomes derived from cetuximab-resistant CRC cells induced drug resistance in cetuximab-sensitive CRC cells. In this study, sera exosomes from CRC patients who did or did not respond to cetuximab and from healthy volunteers were collected. Cetuximab resistance in CRC cells is induced by exosomes collected from patients who do not respond to cetuximab. In contrast, exosomes collected from healthy volunteers promoted the efficacy of cetuximab in CRC cells (Morimura et al., 2018).

### 3.5 Post-transcriptional modification

Post-transcriptional modification is a process in which proteins undergo chemical modifications, such as phosphorylation, ubiquitination, methylation, and acetylation, to perform specific biological functions. Phosphorylation is the most widely studied post-transcriptional modification, and the common protein phosphorylation involved in acquired anti-EGFR resistance in CRC is the activation of the RAF/RAS/MEK/ERK and PI3K/AKT/mTOR signaling pathways (Li et al., 2020). However, other post-transcriptional modifications associated with acquired anti-EGFR resistance in CRC are rarely reported. Lam et al. reported that glycosylation of EGFR at asparagine residue 361 reduces the sensitivity of CRC cells to the anti-EGFR treatment necitumumab by promoting dimerization of EGFR and activation of the EGFR signaling pathway (Lam et al., 2024). A study by Yang et al. revealed that STAT1 was highly expressed in CRC cells resistant to cetuximab. Moreover, insufficient Smurf1-mediated ubiquitination of STAT1 leads to increased proliferation, migration, and invasion of CRC cells, as well as insensitivity to cetuximab (Yang et al., 2024). Rodrigues et al. reported that terminal ⍺2,6-sialylation of EGFR downregulated EGFR expression and suppressed the activation of EGFR, which further increased resistance to cetuximab in CRC cells (Rodrigues et al., 2021). These studies highlight that post-transcriptional modifications, in addition to widely studied phosphorylation, play important roles in acquired resistance to anti-EGFR treatment in CRC cells, which could serve as a potential strategy to reverse this resistance.

## 4 Biomarkers for predicting acquired anti-EGFR resistance

In clinical practice, the prediction of treatment outcomes is critical for improving the management of patients with advanced CRC. Identifying patients with possible acquired resistance to anti-EGFR treatment and the potential mechanism of resistance could guide personalized treatment in patients with advanced CRC.

### 4.1 Tumoral biomarkers for predicting acquired anti-EGFR resistance

Acquiring tumor tissues via biopsy is a common sampling strategy for investigating biomarkers for predicting acquired anti-EGFR resistance. Using this method, genetic mutations in RAS, BRAF, and PIK3CA; amplifications in HER2 and MET; and rearrangements in neurotrophic tyrosine receptor kinase (NTRK)/ROS proto-oncogene 1 (ROS1)/anaplastic lymphoma kinase (ALK)/RET proto-oncogene have been associated with the acquisition of anti-EGFR resistance (Ciappina et al., 2025).

In recent years, researchers have explored novel biomarkers for predicting acquired anti-EGFR resistance in patients with RAS wild-type CRC. For example, Cardone et al. included 136 patients with RAS wild-type CRC who received first-line anti-EGFR therapy. AXL positivity in tumor tissues was associated with worse PFS (6.2 months in the AXL-positive cohort vs. 12.1 months in the AXL-negative cohort) and OS (23.0 months in the AXL-positive cohort vs. 35.8 months in the AXL-negative cohort) (Cardone et al., 2020). Martini et al. reported that in tumor tissues from 82 patients with RAS wild-type CRC who received FOLFIRI plus cetuximab, the EPHA2 tyrosine kinase receptor was detected in 55 tissue samples, and a high level of EPHA2 was associated with a worse PFS (8.6 vs. 12.3 months) and progression rate (29% vs. 9%) (Martini et al., 2019).

Noncoding RNAs also possess the potential to predict acquired anti-EGFR resistance in patients with advanced CRC. Anandappa et al. collected 91 core biopsies from 45 patients with metastatic CRC before single agent anti-EGFR treatment. *In situ* hybridization was used to detect miRNA-31-3p in these biopsies; the results revealed that patients with low expression of miRNA-31-3p exhibited an improved response to anti-EGFR antibody, as well as prolonged PFS and overall survival (OS), before and after adjustment for age, sex, and the sidedness of CRC. Moreover, the expression of miRNA-31-3p does not vary after anti-EGFR treatment (Anandappa et al., 2019). However, Boisteau et al. reported that miRNA-31-3p detected in tumor biopsies was not associated with treatment response in patients with right-sided metastatic CRC who received chemotherapy plus anti-EGFR antibody. Nevertheless, prolonged OS was found in patients with low levels of miRNA-31-3p compared with those with high levels of miRNA-31-3p (Boisteau et al., 2022). Since slight differences exist between these two studies, the prognostic value of miRNA-31-3p should be further verified. Fiala et al. retrospectively enrolled 46 patients with metastatic CRC who were treated with cetuximab or panitumumab and detected the levels of miRNA-125b, let-7c, miRNA-99a, miRNA-17, miRNA-143, and miRNA-145 in tumor biopsies. A high level of miRNA-125b was associated with a better ORR, high levels of miRNA-125b and let-7c, as well as low levels of miRNA-17, were associated with a better disease control rate (DCR), and a high level of miRNA-125b was associated with improved PFS and OS (Fiala et al., 2020). Peng et al. used a lncRNA array to identify potential lncRNAs associated with acquired anti-EGFR resistance in patients with advanced CRC. Nine of the 212 lncRNAs were differentially expressed between patients who achieved disease control and those without a treatment response after anti-EGFR treatment. Among the nine lncRNAs, 5 were associated with PFS, and the lncRNA POU5F1P4 was confirmed to be downregulated in CRC cells resistant to cetuximab (Peng et al., 2018).

### 4.2 Circulating biomarkers for predicting anti-EGFR resistance

Liquid biopsy is a noninvasive biospecimen collection method that involves the collection of blood samples for various tumor indicators, including cell-free DNA (cfDNA), circulating tumor DNA (ctDNA) and microRNAs (miRNAs). In particular, cfDNA analysis can detect multiple mechanisms of coexisting drug resistance that may be missed by single-focus tumor tissue biopsies, making it an advantageous assay for assessing tumor heterogeneity (Blakely et al., 2017). The latest studies have utilized liquid biopsy-based ctDNA and cfDNA to predict treatment response and survival in CRC patients receiving anti-EGFR antibody-based treatment, further contributing to the personalized management of CRC. For example, the CAPRI-2 GOIM trial included 192 patients with metastatic CRC harboring wild-type RAS and planned to receive FOLFIRI plus cetuximab. According to the results of baseline liquid biopsy-comprehensive genomic profiling of potential acquired anti-EGFR resistance genes (including RAS, BRAF, EGFR, PIK3CA, MAP2K1, MET, RET, ALK, ROS1, NTRK, NFR, and FGFR mutations, as well as HER2 amplification) in ctDNA with FoundationOne (F1) CDx and F1 Liquid (F1L) CDx (324 genes), patients were stratified into a wild-type group and a mutation group. The ORR was 54.5% for the mutation group and 78.1% for the wild-type group. The median PFS was 8.68 months for the mutation group compared with 12.35% for the wild-type group (Ciardiello et al., 2025). The PLATFORM-B study included 100 patients with metastatic CRC harboring wild-type RAS and who were scheduled to receive first-line chemotherapy plus cetuximab. ctDNA at baseline and early after treatment was analyzed with next-generation sequencing, and mutations in RAS, MEK, and BRAF were negatively correlated with PFS. Moreover, a decrease in mutations in RAS, MEK, and BRAF after treatment was associated with increased response and PFS (Vidal et al., 2023). Toledo et al. enrolled 25 patients with metastatic CRC harboring wild-type RAS and who received first-line FOLFIRI plus cetuximab. A 2-year follow-up was conducted, during which a routine sample was collected for cfDNA detection via the BEAMing technique. Wild-type KRAS, NRAS, PIK3CA, and BRAF are associated with prolonged treatment response, whereas mutations in these genes are associated with acquired resistance (Toledo et al., 2017). These studies highlight the potential of liquid biopsy as a tool for predicting treatment response and survival in patients with CRC harboring wild-type RAS and receiving anti-EGFR antibody-based treatment. When therapeutic regimens are formulated to reverse acquired drug resistance, liquid biopsy may be useful because patients may have multiple mechanisms of resistance, and regular identification of cfDNA is critical for continued treatment after resistance. Second, the ease of accessibility, low invasiveness, and ease of monitoring of liquid biopsies make them an even more attractive testing technique.

Although noncoding RNAs from tumor biopsies have potential predictive value for anti-EGFR resistance, the process of acquiring tumor biopsies could cause harm to patients. On the other hand, circulating RNAs, despite easy and harmless collection of samples, can be degraded by RNAse, resulting in inaccurate prediction. Exosomes, however, can protect RNAs from degradation because of their bilayer structure. Studies have reported that noncoding RNAs derived from exosomes have good predictive value in cancer patients (Zhu et al., 2024; Jing et al., 2024; Linares-Rodriguez et al., 2025). However, relevant studies have rarely been conducted on anti-EGFR resistance in CRC. Yang et al. included 53 patients with advanced CRC who received cetuximab treatment and collected blood samples for exosome separation. Exosomal lncRNA urothelial carcinoma-associated 1 was upregulated in patients with poor treatment response compared with those with objective response (Yang et al., 2018).

## 5 Clinical evidence of coping with acquired anti-EGFR resistance in patients with CRC

In recent years, researchers have devoted tremendous effort to coping with anti-EGFR resistance in patients with advanced CRC (Table 1). Since acquired anti-EGFR resistance is associated with resistance after treatment with anti-EGFR antibodies, the treatment strategies discussed here are all second-line or above options. The common strategy is to identify the molecules associated with acquired anti-EGFR resistance and then add drugs targeting these molecules to improve the treatment response. For patients with BRAF mutations, the combination of a BRAF inhibitor with an anti-EGFR antibody is a common strategy. For example, Yaeger et al. conducted a pilot study and reported that among 15 patients with BRAF-mutant CRC who received the BRAF inhibitor vemurafenib and the anti-EGFR antibody panitumumab, the ORR was 13%, the median PFS was 3.2 months, and the median OS was 7.6 months (Yaeger et al., 2015). Tan et al. treated 32 BRAF-mutant CRC patients with vemurafenib combined with the EGFR inhibitor erlotinib and reported that the ORR was 32% (Tan et al., 2023). Tabernero et al. reported that after treatment with the BRAF inhibitor encorafenib and cetuximab, patients with BRAF-mutant CRC had an ORR of 22% and a median PFS of 4.2 months (Tabernero et al., 2016). The phase 3 BREAKWATER trial compared the efficacy of encorafenib + cetuximab + chemotherapy with that of the standard of care in patients with naïve mCRC harboring BRAF mutations (Kopetz et al., 2025). This trial reported that one of the primary endpoints of the ORR was 60.9% in the combination treatment group compared with 40.0% in the standard-of-care group. However, the trial is still ongoing, and the survival data are premature. Another common strategy for coping with BRAF mutation-induced resistance is to further inhibit downstream factors of BRAF, such as PI3K and MEK. In the study of Tabernero et al., another cohort of patients received encorafenib, cetuximab, and the PI3Kα inhibitor ALP. The ORR was 27%, and the median PFS was 5.4 months in this cohort (Tabernero et al., 2016). Corcoran et al. reported that the combination of the BRAF inhibitors dabrafenib and panitumumab and the MEK inhibitor trametinib achieved an ORR of 21% in patients with BRAF-mutant CRC (Corcoran et al., 2018). In the BEACON CRC trial, 224 patients with BRAF-mutant metastatic CRC received encorafenib plus cetuximab and the MEK inhibitor binimetinib. The ORR was 26.8%, and the median OS was 9.3 months in these patients (Tabernero et al., 2021).

**TABLE 1:** Clinical evidence of treatment strategies to cope with anti-EGFR resistance in CRC patients.

Study	Mechanisms associated with anti-EGFR resistance	Coping strategy	Outcomes
Yaeger et al. (2015)	BRAF mutation	Vemurafenib + panitumumab	ORR: 13% Median PFS: 3.2 months Median OS: 7.6 months
Tan et al. (2023)	BRAF mutation	Vemurafenib + erlotinib	ORR: 32%
Tabernero et al. (2016)	BRAF mutation	Encorafenib + cetuximab	ORR: 22% Median PFS: 4.2 months
		Encorafenib + cetuximab + ALP	ORR: 27% Median PFS: 5.4 months
Corcoran et al. (2018)	BRAF mutation	Dabrafenib + panitumumab + trametinib	ORR: 21%
Tabernero et al. (2021)	BRAF mutation	Encorafenib + cetuximab + binimetinib	ORR: 26.8% Median OS: 9.3 months
Rimassa et al. (2019)	MET overexpression	Tivantinib + cetuximab	ORR: 9.8% Median PFS: 2.6 months Median OS: 9.2 months
Strickler et al. (2021)	MET overexpression	Cabozantinib + panitumumab	ORR: 16% Median PFS: 3.7 months Median OS: 12.1 months
Van Cutsem et al. (2014)	Hyperactivation of IGF-1R	Ganitumab + panitumumab	ORR: 22% Median PFS: 5.3 months Median OS: 10.6 months
Sclafani et al. (2015)	Hyperactivation of IGF-1R	Dalotuzumab + cetuximab	Median PFS: 5.6 months Median OS: 17.9 months
Yaeger et al. (2024)	RAS mutation	Adagrasib + cetuximab	ORR: 34% DCR: 85.1% Median PFS: 6.9 months Median OS: 15.9 months

Abbreviations: EGFR, epidermal growth factor receptor; CRC, colorectal cancer; IGF-1R, insulin-like growth factor-1, receptor; ORR, objective response rate; DCR, disease control rate; PFS, progression-free survival; OS, overall survival.

For patients with MET overexpression-induced anti-EGFR resistance, the combination of an anti-EGFR antibody with a MET inhibitor is a potential strategy to address resistance. Rimassa et al. evaluated 41 patients with metastatic CRC harboring MET overexpression who were treated with cetuximab plus tivantinib, a MET inhibitor. The ORR was 9.8%, the median PFS was 2.6 months, and the median OS was 9.2 months (Rimassa et al., 2019). Strickler et al. treated patients with panitumumab and cabozantinib (a multiple kinase inhibitor with a c-MET inhibitory effect) and reported that the ORR was 16%, the median PFS was 3.7 months, and the median OS was 12.1 months (Strickler et al., 2021).

For patients who acquire resistance due to hyperactivation of IGF-1R, the addition of inhibitors targeting IGF signaling is theoretically feasible. However, a clinical study revealed no tremendous benefits of an IGF-1R inhibitor (ganitumab) plus panitumumab compared with panitumumab alone. This study compared the treatment response and survival of patients in the panitumumab plus ganitumab arm and the panitumumab plus placebo arm. The ORRs were 22% and 21%, the median PFS times were 5.3 months and 3.7 months, and the median OS durations were 10.6 months and 11.6 months (Van Cutsem et al., 2014). Sclafani et al. reported that in advanced CRC patients with high expression of IGF-1, weekly dalotuzumab (an anti-IGF-1R antibody) plus cetuximab only showed numerically better PFS (5.6 vs. 3.6 months) and OS (17.9 vs. 9.4 months) than did the combination of cetuximab plus placebo (Sclafani et al., 2015).

For anti-EGFR resistance due to RAS mutation, the addition of RAS inhibitors is a potential strategy. Yaeger et al. reported that in patients with acquired anti-EGFR resistance due to RAS mutation, the combination of cetuximab plus adagrasib (an irreversible KRAS inhibitor) yielded an ORR of 34%, a DCR of 85.1%, a median PFS of 6.9 months, and a median OS of 15.9 months (Yaeger et al., 2024). However, this regimen is usually applied in patients refractory to chemotherapy and harboring RAS mutations (Fakih et al., 2023; Kuboki et al., 2024; Desai et al., 2024). Therefore, the potential of RAS inhibitors plus anti-EGFR antibodies in patients with acquired anti-EGFR resistance due to RAS mutations should be further investigated.

The MSI-H/dMMR status is another factor leading to anti-EGFR resistance but also enhances the antitumor immune response. As a result, the addition of ICIs is a potential strategy for this purpose. Studies have reported that the combination of ICIs and anti-EGFR antibodies has good efficacy in patients with head and neck cancer (Tao et al., 2023; Yang et al., 2022; Sacco et al., 2019). In CRC, one study is ongoing to explore the efficacy of ICIs plus anti-EGFR antibodies in patients with MSI-H/dMMR and BRAF mutations (Elez et al., 2024). Other available studies have investigated this regimen in patients with microsatellite stable/proficient mismatch repair status or regardless of patients’ microsatellite/mismatch repair status. These studies reported an ORR of 2.6%–33%, a median PFS of 4.1–7.3 months, and a median OS of 15.1–17.4 months (Xu et al., 2024; Quan et al., 2023; Fountzilas et al., 2021). However, the role of ICIs plus anti-EGFR antibodies in patients with acquired anti-EGFR resistance is still unclear. The optimal treatment strategy involving this regimen should be further verified in clinical trials.

## 6 Limitations

Although the mechanisms of acquired anti-EGFR resistance have been partly illustrated, the actual conditions may be more complicated in clinical practice. In the same patient, several resistance mechanisms may coexist. There is still a long way to go to find all drug-resistant mutations accurately and to determine their interactions. Under the requirements of precision medicine, the prediction of patient prognosis is critical. Although biomarkers from both tumoral biopsy and liquid biopsy have potential for predicting patient prognosis, most studies lack a sufficient sample size and integrated validation.

In terms of strategies to reverse anti-EGFR resistance, treatments that target the mechanisms of acquired anti-EGFR resistance present potential efficacy. However, most of the studies have common limitations that hamper the quality of evidence, such as small sample sizes and single-armed study designs. As a result, the efficacy of available corresponding treatments for the mechanisms of acquired anti-EGFR resistance should be further verified by studies with larger sample sizes and well-designed randomized, controlled trials. Moreover, for specific patient subgroups, such as patients with HER2 amplification or ARID1A mutation, there is currently no effective targeted therapy to reverse acquired anti-EGFR resistance, highlighting the gap between the knowledge of the molecular mechanisms of anti-EGFR resistance and clinical treatment strategies to reverse this issue. The current review also has several limitations, such as a lack of data synthesis and heterogeneity among studies.

## 7 Conclusion

**FIGURE 1:**
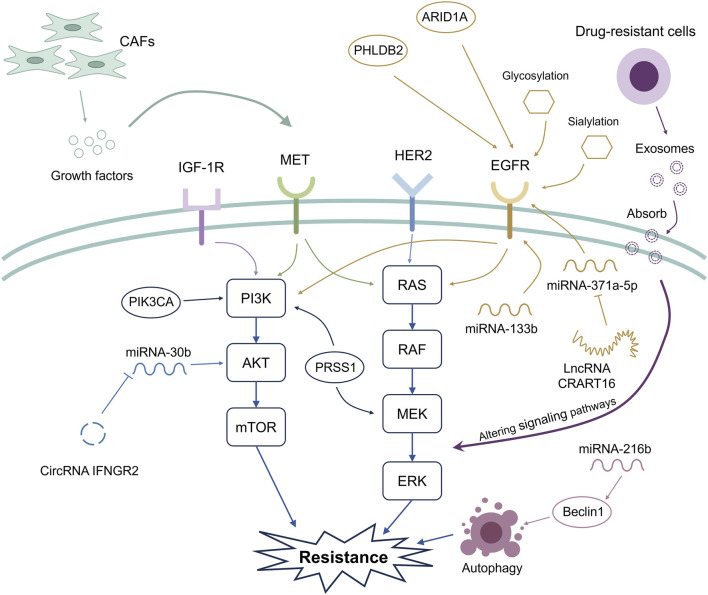
Mechanisms related to acquired anti-EGFR resistance. The widely recognized mechanisms that induce acquired anti-EGFR resistance are associated with the activation of the PI3K/AKT/mTOR and RAS/RAF/MEK/ERK signaling pathways. Novel mechanisms associated with acquired anti-EGFR resistance are also being discovered. These include microsatellite stability/mismatch repair functions, noncoding RNAs, the tumor microenvironment, exosome-mediated intracellular communication, and post-transcriptional modification. CAFs can release growth factors, contributing to the activation of signaling pathways associated with acquired anti-EGFR resistance. Additionally, genetic mutation of ARID1A and upregulation of PHLDB2 activate EGFR, thus leading to acquired anti-EGFR resistance.

Acquired resistance to anti-EGFR treatment is a critical factor for worsening the prognosis of CRC patients harboring wild-type RAS. The mechanisms that lead to acquired anti-EGFR resistance are very complex, and new mechanisms are being discovered (Figure 1). Whether the corresponding treatments could improve the clinical management of patients with acquired anti-EGFR resistance deserves further investigation. Moreover, novel treatment strategies to cope with acquired anti-EGFR resistance, such as bispecific antibodies, SHP2 inhibitors, combinations of EGFR inhibitors with cyclin-dependent kinase 4/6 inhibitors, death receptor-5-targeted nanocarriers, and novel anti-EGFR antibodies with distinct binding modes, are being investigated (Stuber et al., 2021; Sorokin et al., 2022; Zhou et al., 2025; Vijayaraghavan et al., 2020; Liu and Chen, 2025). In addition, several ongoing clinical studies may identify future directions for this area, such as further investigations into the prediction of acquired anti-EGFR resistance with ctDNA and the potentiation of anti-EGFR antibodies with novel therapeutic agents (NCT05051592, NCT06714357, and NCT03263663).
